# Treatment of Brachial Plexus Injuries following Gunshot Injuries: A Systematic Review

**DOI:** 10.1155/2024/7708192

**Published:** 2024-08-30

**Authors:** Rohun Gupta, Isabel Herzog, Lauren Phung, Jacquelyn Roth, Joseph Weisberger, Margaret Luthringer, Edward S. Lee, Ashley Ignatiuk

**Affiliations:** ^1^ Division of Plastic and Reconstructive Surgery St. Louis University School of Medicine, St. Louis, MO, USA; ^2^ Department of Plastic Surgery Rutgers New Jersey School of Medicine, Newark, NJ, USA; ^3^ Oakland University William Beaumont School of Medicine, Rochester, MI, USA

## Abstract

**Introduction:**

Brachial plexus injuries (BPI) from gunshot injuries are uncommon but usually severe and can cause chronic pain, loss of function, and permanent nerve damage. Multiple surgical techniques including neurolysis, end-to-end suture repair, and graft repair have been described for the treatment of these injuries. However, surgical indication, timing, and technique for these injuries remain controversial. This systematic review aims to investigate the treatment modalities for patients with BPI due to gunshot-related injuries.

**Methods:**

The Preferred Reporting Items for Systematic Reviews and Meta-Analysis (PRISMA) methodology was employed for this review. PubMed, Cochrane Reviews, Embase, and CINAHL databases were included. The following keywords constituted our search criteria: gun-shot-wounds, brachial plexus, traum^∗^, and management.

**Results:**

A total of 90 studies were imported for screening, from which 9 papers met our final inclusion/exclusion criteria. The most common studies utilized in this review were retrospective chart reviews followed by case series. In total, there were 628 patients that suffered from gunshot wounds to the brachial plexus. Most patients underwent some form of delayed nerve repair consisting of neurolysis, end-to-end epineural repair, or graft repair with a sural or antebrachial cutaneous nerve graft. Several patients suffered from complications, with neuroma being the most common long-term complication that required reoperation.

**Conclusion:**

The optimal timing for surgeries involving BPIs should be determined after examining the level of nerve damage, associated injuries, operative risks, and electrophysiological workup for indications of spontaneous regeneration. Early surgical interventions were indicated for patients presenting with associated vascular or thoracic injuries, compressive masses, and nerve transection by sharp instruments in most selected papers.

## 1. Introduction

In the United States, intentional firearm-related injuries are the seventh highest nonfatal violence-related injury [1]. Patients who suffered from gunshot trauma commonly encounter chronic pain, sensation loss, neuropathic pain, and motor dysfunction [2–8]. In addition to the directly inflicted trauma, both high- and low-velocity gunshot injuries induce a shockwave and cavitation: leading to considerable soft tissue stretching, compression, and shearing [2, 8–11]. Furthermore, this can lead to a wide range of neurological sequela including neuropraxia, axonotmesis, or neurotmesis injuries [8, 9].

Although traumatic brachial plexus injuries (BPI) are relatively uncommon, they are typically the most difficult injuries to treat with peripheral nerve surgery [12]. GSWs comprise between 3% and 12% of BPIs, and some studies have found that GSWs are the second leading cause of BPIs, preceded only by traction injuries [4, 13]. Unlike other countries, the United States has not experienced a decline in firearm-related deaths in recent decades; the number nonfatal gunshot wounds (GSW) rose by 26% between 2014 and 2017—approximately twice the number of gun violence deaths [2, 14]. In addition, a study by Secer et al. found that 36% of their patients who had suffered from GSW-related BPI had coexisting injuries to the vascular structures, soft tissue, and musculature [9, 15]. Despite the increase in incidence and severity of injury, there is a lack of reliable methods to distinguish between neuropraxia, axonotmesis, or neurotmesis injuries through clinical examination. Though neuropraxia is considered to be common, some literature suggests a high incidence of nerve laceration after GSWs [11].

Timing of neurological intervention for GSW-related BPI is an issue of great debate, second to the uncertainty reported in the literature. Typically, studies have suggested that clean wounds without infection, stable fractures, and restoration of compromised vascular issues are priorities and grounds for delayed nerve repair [16]. Additionally, early surgical revision may not be indicated as there may be spontaneous recovery along with the difficulties in assessing extent of nerve damage [17]. Studies by Omer have advocated for late intervention, as several patients tend to have spontaneous return of function [18, 19].

On the other hand, several other studies have determined that early surgical intervention leads to optimal patient outcomes due to a limited amount of scar tissue and ease of identifying nerves [15, 20, 21]. Further, delayed exploration of completely lacerated nerves may result in worsened clinical outcomes [11]. The surgical techniques of neurolysis, direct repair, and nerve graft for treatment of GSWs involving BPIs have been described in the literature [22]. Still, time to surgical intervention for patients suffering from gunshot injuries to the brachial plexus is controversial due to the inability to clinically assess which patients require early surgical intervention compared to those that do not.

To the best of our knowledge, there has not been an informative review that has evaluated outcomes of patients that have undergone surgical management for brachial plexus injuries stemming from gunshot injuries. Therefore, the purpose of this systematic review is to assess the treatment modalities and time to treatment for patients with BPI due to gunshot-related injuries with consideration of indication, timing, technique, and outcomes.

## 2. Methods

The design, execution, and documentation of this review followed the Preferred Reporting Items for Systematic Reviews and Meta-Analyses (PRISMA) guideline. The review was registered with the PROSPERO database system [23–25].

### 2.1. Search Strategies

To identify articles on gunshot injuries to the brachial plexus and nerve repair, a specific search strategy was developed using the following keywords: ((manag^∗^) OR (management)) AND ((brachial) OR (brachial plexus)) AND ((gunshot wound) OR (gunshot) OR (GSW)). The databases used for this search included PubMed, COCHRANE, EMBASE, and CINAHL. No restrictions were applied regarding publication date, language, or study type.

### 2.2. Study Selection

The identified articles were uploaded to COVIDENCE, an online tool for initial screening and data extraction. Duplicates were removed, and studies were selected based on criteria that included English language only, exclusion of systematic reviews, and a focus on gunshot injuries to the brachial plexus. Two independent reviewers (RG and JW) conducted the screening of titles and abstracts, with a third reviewer (ML) resolving any disagreements. After filtering out irrelevant studies, the remaining papers underwent a full-text review by the same two independent reviewers, with any conflicts again being resolved by the third reviewer (ML).

### 2.3. Quality Assessment

The quality of the selected manuscripts was evaluated using the Methodological Index for Non-Randomized Studies (MINORS) guidelines. This guideline consists of 12 questions which assess the manuscripts for clarity of stated aim, appropriate endpoints relative to the stated aim, unbiased endpoint assessment, prospective collection of data, inclusion of consecutive patients, appropriate follow-up period, less than 5% loss to follow-up, prospective study size calculation, presence of an adequate control group, presence of contemporary groups, baseline equivalence between the groups, and sufficient statistical analyses [26]. Each criterion has a maximum value of 2 points, resulting in a maximum score of 16 and 24 for noncomparative studies and comparative studies, respectively. Three reviewers (RG, JW, and ML) independently evaluated each study. The scores from the reviewers were averaged or each paper for a final score which was rounded to the nearest whole number.

### 2.4. Data Extraction

Data was extracted from eligible studies independently by two authors (RG and JW). A standardized extraction was used to obtain the following information:

1. Methods: study type, level of evidence, and sample size
2. Participants: year of study, sample size, age of participants, gender, and procedural indication
3. Intervention: the technique used, details of the procedure, and follow-up period
4. Outcomes: postprocedural complications, reoperations, aesthetic outcome, patient satisfaction, and long-term complications

### 2.5. Data Analysis

Once fully extracted, the data were imported into Microsoft Excel (Microsoft Office Excel 2022 Redmon, WA, USA). Within Microsoft Excel, the data were compiled and organized into tables for use in the manuscript.

## 3. Results

### 3.1. General Overview

**Figure 1:**
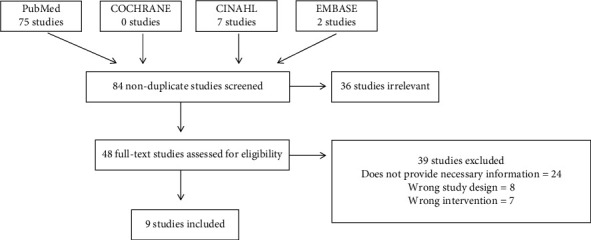
Study selection based upon PRISMA-P guidelines.

Figure 1 shows the study selection PRISMA flow diagram. A total of 90 studies were imported for screening, from which 6 duplicates were removed. 84 studies were screened, and 36 studies were screened as irrelevant. 48 full-text studies were assessed, and 9 papers met our final inclusion/exclusion criteria (Figure 1). Quality assessment was performed on the selected studies (Table 1). A meta-analysis was not able to be performed owing to the lack of control groups in the included studies.

### 3.2. Clinical Characteristics Analysis

Nine studies were utilized in total for the clinical characteristic analysis. Of these 9, 6 were retrospective chart reviews and 3 were case series (Table 1). The number of patients varied amongst the 9 studies, ranging from 1 to 159 patients (Table 2). The aggregate data determined that there was a total of 628 patients, with the majority of patients (67.7%) being male. The patient age range was considerable, spanning from 4 to 70 years.

Extent of brachial plexus injury was found to be at the root to trunk, division to cord, and cord to nerve levels. There were two studies that specified the number of patients with each type of brachial plexus injury [27, 28].

Between the two studies, it was determined that 30 patients (22.1%) had root to trunk injuries, 39 patients (28.7%) had division to cord injuries, and 67 patients (49.2%) had cord to nerve injuries. Associated vascular injuries were noted in two studies with a combined total of 46 out of 121 patients (38.0%) who required some degree of vascular repair due to gunshot injury to the brachial plexus [26, 27]. Additional injuries requiring intervention included chest tube placement, thoracotomy, pseudoaneurysm excision, and sympathectomy.

Procedures that were conducted included neurolysis, end-to-end epineural repair, graft repair, or a combination of the prior three procedures mentioned. All studies utilized autograft, with either sural nerve or antebrachial cutaneous nerve as the donor [22, 27–29]. An anterior approach was utilized for most patients with some patients undergoing a posterior subscapular approach if the gunshot wound involved the plexal spinal nerves proximal to the spine, at an intraforaminal level, or if the lower roots or trunks were affected. In addition, a study by Dubuisson and Kline also reported utilizing a posterior approach if there was presence of heavy scar tissue anteriorly [30]. Patients underwent procedures 1.5 to 24 months after initial injury with one paper by Kim et al. stating that some patients underwent nerve repair procedures earlier due to concomitant vascular or other severe injuries that were present [22].

Complications were noted in all included studies except for two by Kim et al. and Samadian et al. [22, 31]. The most common acute complications were infection, hematoma, and thrombosis. Long-term complications included neuroma formation and varying degrees of motor or sensory loss in the affected nerves. Several patients underwent reoperation due to sensory and mass effect due to neuroma formation. Follow-up length varied between all the studies with the range being from 0 to 46 months.

## 4. Discussion

Most GSW-related BPIs causing neurological damage result from direct bullet trauma and indirect heat and shock wave to neighboring tissue [32]. GSWs, unlike other traumatic injuries, tend to have additional unforeseen injuries. Kim and Leopold determined that surface injuries do not necessarily reflect the potential for deep tissue damage, necrosis, and viability [33]. Arunkumar et al. state that damage from gunshot injuries are two-fold; there is primary damage consisting of bullet trajectory, which includes shearing and compressive forces, and secondary damage from kinetic injury, which leads to damage outside of the bullet's tract [34, 35]. Additionally, nerve elements are seldom damaged by nerve impact but rather forces of shock leading to cavitation, compression, and stretching [36]. Furthermore, this is theorized to lead to damage outside of the path of projectiles and longer nerve segments [36]. The neurological impact of cavitation is broad, spanning from neuropraxic stretch injuries to nerve laceration [37]. The broad spectrum of possible injuries and no reliable method to distinguish between axonotmesis, neuropraxia, and neurotmesis makes it extremely difficult to time treatment in patients [11].

Ideal timing of treatments for patients with BPI remains controversial, as it depends on a multitude of factors. Prior to nerve reconstruction, it is important that the patient possesses an uninfected clean wound with soft tissue coverage, stable fracture, and suitable limb perfusion [3, 9]. Surgeons operating on nonfatal GSWs may need to perform multiple procedures, such as irrigation and debridement, operative fracture fixation, and amputation based on the extent of inflicted soft tissue injuries [14].

Based off our dataset, we have defined immediate intervention as occurring 0–1.5 months from injury, early intervention as 1.5–6 months from injury, and delayed as >7 months from injury. One study by Secer et al. reported occasionally performing immediate interventions for patients if nerve elements were found to be totally transected [15]. Other studies stated that immediate intervention may be indicated if there are concomitant vascular or thoracic injuries, compressive masses, or nerve transections [22, 27–31]. All studies included in this review reported performing early intervention for the majority of their patients at 4 to 4.75 months from injury. In these studies, the delay in surgical intervention was often attributed to potential spontaneous regeneration measured by electrophysiological studies for neurological deficits [9, 27, 28, 31]. Kline, in his 1989 publication, determined incomplete recovery through clinical exam, while Kim et al. utilized NAP testing to assess for neuropathic recovery [22, 27]. Other studies by Moore et al. have stated that electrodiagnostic studies starting at 10 weeks from injury complemented by serial physical examinations are critical in assessing recovery, as degree of neuropathic injury and information regarding spontaneous recovery can be monitored [38]. Repair in these studies consisted of varying degrees of reconstruction composed of neurolysis, nerve transfers, or end-to-end repair of lacerated nerves. There were some studies that reported performing neuroma resection followed by nerve repair [15].

Moreover, delayed surgical intervention was also observed in patients who ultimately demonstrated incomplete recovery in the initial conversative approach [22, 27, 29, 31]. There was one study by Samadian et al. who advised against delaying surgical treatment of more than one year postinjury in cases with complete neurological deficits and absence of spontaneous recovery over 3 months from the initial neurological insult [31]. Furthermore, other studies have also found that nerve repair should occur after 3 months, as there is often complete spontaneous resolution of clinical symptoms [27, 39, 40]. Additionally, Samardzic et al. determined that surgical repair should only occur if there has been complete functional loss in one or more distributions for a time greater than 3 months and that surgery should not be delayed for greater than 1 year after injury [36, 41, 42]. However, glial scarring and neuroma formation from the insult may further complicate treatment decisions [11]. The likelihood of spontaneous recovery must be considered, so early surgical exploration may not always be best; however, assessing the extent and severity of the nerve damage is difficult [9, 22].

Both complex region pain syndrome (CRPS) and non-CRPS neuropathic pain complicate brachial plexus injuries and play a role in determining management [9, 15, 22, 29]. Noncausalgic pain refractory to conservative management with medications and physical therapy is considered an indication for operative intervention [2, 15]. Neurolysis of lower elements, while minimally helpful with functional recover, often yielded pain relief [9, 15]. Consequently, for injuries to C8 or T1 spinal nerves, lower trunk, or medial cord lesions, intractable pain is the chief determining factor for operative intervention [15, 22]. Given the initial attempt with extensive medical management, the studies suggest that operative intervention was often delayed for several months [15, 22, 31]. Precise timing of operative intervention, as determined by nonfunctional indications, was not examined in the studies assessed.

The role imaging in brachial plexus injury management, while also largely unexplored in the studies reviewed, warrants additional attention as advancements in recent years have improved visibility into both timing and appropriate intervention for peripheral nerve injuries. Modalities including MR neurography and high-resolution echography are increasingly used to investigate nerve viability, offering improved sensitivity and detail compared with conventional electroneurography and neurosonography evaluations [43, 44, 47]. MR neurography enables earlier and more precise classification of location, type, and extent of nerve damage, particularly for preganglionic lesions, while high resolution ultrasound offers enhanced assessment of postganglionic lesions [45, 46]. These modalities afford insight into the likelihood of spontaneous recovery and, if required, the time sensitivity of intervention [43, 47].

Analysis of selected papers provides the rationale in selecting surgical procedures based on the level of injury, microscopic, and intraoperative findings. In two included studies, it was found that adult patients with lesions at lower roots, lower trunks, or medial cord to ulnar nerve may be treated conservatively due to low yield of functional recovery from surgical repair unless unretractable pain is indicated [22, 31]. Although there is limited literature that may explain these findings, it may be secondary to a greater distance to target muscles from the lower portions of the brachial plexus leading to motor endplate degeneration. Still, nerve transfers can be a surgical option in several conditions including when there is a proximal injury with a significant distance to the target muscle and when there have been prior failed interventions for proximal nerve repair [48]. Nerve transfers, used primarily for preganglionic root injuries, may be extraplexus or intraplexus, with options to utilize intact nerve roots [49]. Utilization of nerve transfers for brachial plexus injuries is a successful option, with reports of 80–90% of patients experiencing significant improvements after a procedure [50]. However, a study by Hems reports that potential problems with nerve transfers include preexisting damage to donor nerves, which may result in a variation of patient outcomes [51]. All in all, the use of nerve transfers has widened the scope of reconstructive options in patients suffering from brachial plexus injuries and must be weighed heavily on an individual basis.

Lesions in continuity with positive nerve action potential (NAP) during intraoperative electrical stimulation treated with only external neurolysis demonstrated successful functional outcomes in a high percentage of cases. [22, 30, 31, 52] Meanwhile, resection and nerve reconstruction were selected for lesions in continuity with negative NAP which resulted in at least Grade 3 of the LSUHSC motor and sensory system in many patients [27, 29]. Although time from injury to neurolysis was not specified in the selected papers of this review, a study by Bage and Power has stated that in the additional presence of neuropathic pain, early neurolysis may lead to drastic improvements in a patient's functional and clinical symptoms [53]. Still, there are a limited number of retrospective and prospective studies that have looked at the association between early neurolysis and neuropraxic injuries, which may demonstrate that early neurolysis has the potential to enhance recovery in neuropraxic injuries.

When graft repair was indicated, the sural nerve was commonly used followed by antebrachial cutaneous nerve graft, with satisfactory results reported between 50% and 65% of cases [22, 30, 31]. The graft length reported from studies ranges from 1 cm to 6.5 cm [9, 22, 29, 30]. Several studies reported reoperation due to neuroma formation. Often, nerve reconstruction with autograft or allograft is conducted as very small nerve gaps following neuroma removal is rare [54]. Eberlin et al. and Souza et al. found that in their experience, patients opted for allograft as there may be a risk of neuropathic pain or neuroma formation at the donor site [54, 55]. Still, Sosin et al. state that recommendations for utilizing autograft rather than allograft are dynamic, and instead, providers should use clinical judgement based on history and surgical site to decide which intervention is best suited for the patient [56].

Secer et al. conducted a statistical analysis on the relationship between neural graft length and clinical outcomes. Although the result was deemed to be statistically insignificant, this study concluded the potential correlation between shorter graft length and better surgical results, which should be examined in future studies [9]. Level of injury, intraoperative findings, nerve conduction studies, and type of surgical treatments are emphasized as salient prognostic factors of patients' clinical outcomes across selected studies in our report. Despite this, we found no correlation between the level of injury and the timing of treatment in our study.

There are several limitations in our review. A major limitation was the inclusion of only English studies in our search criteria. While this methodology is consistent with many systematic reviews, it limits the scope of information accessible from both a patient and management perspective. Inclusion of non-English publications may have both closed information gaps observed in the literature reviewed such as the role of imaging in operative intervention and the timing of pain-driven operative interventions, as well as reduced reliance on any one publication for data. For example, there is considerable overlap in the patients and criteria employed by both Dubuisson and Kim. Moreover, several included studies rely on the work of Klein in their analysis, limiting the ability to assess findings independent of one another. As such, future studies should prioritize inclusion of non-English publications. In addition, although most of the studies included in this review were retrospective chart reviews, there were still several case series. The presence of case series complicated a comparison of respective treatment efficacies. The development and use of a uniform quality assessment method may allow for more accurate comparison of treatment options between studies. Furthermore, it is critical to consider surgical bias and has the potential to be a limitation that cannot be excluded from this study. Lastly, results and data were presented broadly among studies, which added to the complexity in comparing studies. To address these various issues, it is critical for studies to increase the number of patients and present findings in a more standardized method to further various other findings within this topic.

## 5. Conclusion

The optimal timing of surgical intervention for patients suffering from gunshot injuries to the brachial plexus should be considered after the level of nerve injury, associated injuries, and electrophysiological workup has been conducted. Acute surgical intervention is indicated when there are additional life-threatening injuries that require management. Most patients should look to undergo nerve reconstructive procedures 3 months after initial injury, as there may be spontaneous resolution of clinical symptoms. Surgical interventions for nerve injuries may include end-to-end repair, nerve grafting, and neurolysis.

## Figures and Tables

**Table 1:** Evaluation of quality of included brachial plexus injuries studies.

Study	Study design	Level of evidence^∗^	Indications for surgery	MINORS score	Statistical analysis
Dubuisson and Kline (2002)	Retrospective chart review	III	Gunshot wounds associated with vascular or thoracic injuries, sharp or blunt transection, severe pain syndrome, no recovery in distribution of elements at 2 to 4 months postinjury, closed traction injuries, root avulsion	10	No
Geudes et al. (2020)	Case series	IV	Pain distress and motor deficits	10	No
Iordarche et al. (2021)	Case series	IV	Neuropraxia, axonotmesis, and neurotmesis	8	No
Kim et al. (2004)	Retrospective chart review	III	Complete loss in the distribution of at least one element and no improvement clinically or by EMG in early months postinjury; incomplete loss where pain could not be managed pharmacologically; pseudoaneurysm, clot, or fistula involving the plexus; true causalgia	9	No
Kim et al. (2006)	Retrospective chart review	III	Association with vascular repair or other severe injuries, complete functional and electrophysiological loss after 2 to 4 months postinjury, incomplete loss with uncontrollable pain; plexus compression by pseudoaneurysm, clot, or arteriovenous fistulae; true causalgias; noncausalgic pain associated with injuries to C8 and T1 roots, medial cord, and medial cord-to-ulnar outflow which cannot be controlled pharmacologically	7	No
Kline (1989)	Case series	IV	Persistent complete loss in distribution of one or more elements, severe noncausalgic pain in less complete injuries, pseudoaneurysms, and clot compressing plexus	9	No
Kline and Judice (1983)	Retrospective chart review	III	Complete neurological deficits without signs of regeneration between 2–4 months postinjury (GSW) and 4-5 months postinjury (stretch injury), sharp laceration, and lesions being or potentially being complicated by compressive masses	11	No
Samadian et al. (2009)	Retrospective chart review	III	Association with vascular injury, incomplete recovery of neurological deficit and intractable neurological pain	8	No
Secer et al. (2009)	Retrospective chart review	III	Neurological deficits in the distribution of one or more elements of the plexus without improvements between 6 weeks and 4 months after injury	12	Yes

^∗^Oxford Center for Evidence-Based Medicine—level of evidence for the included studies.

**Table 2:** Overview of included brachial plexus injuries studies.

Study	*n*	Age	Injury	Time from injury to intervention	Surgical procedure	Complications	Follow-up	Conclusion
Dubuisson and Kline (2002)	8	5 years to 70 years	Broad spectrum of brachial plexus injuries	4.25 months	Neurolysis, neurotization, end-to-end repair, sural, and/or antebrachial cutaneous nerve graft	Reoperation, thrombosis	42 months	The decision regarding the best procedure for repair is made when the brachial plexus is exposed and after electrophysiological examinations are conducted
Geudes et al. (2020)	1	16 years	Upper trunk of right brachial plexus	6 months	External neurolysis, sural nerve graft	None provided	7 months	Multidisciplinary management including appropriate surgical treatment, physical, psychological, and occupational therapy along with factors such as time taken from injury to surgery, age of patient, and type of lesion may lead to enhanced recovery
Iordarche et al. (2021)	17	16.4 years to 35 years	Broad spectrum of brachial plexus injuries	3.73 months to 12 months	Tendon transfer, neurotization, neurolysis, sural nerve graft	Infection, reoperation	0 months to 8 months	Due to the unique experience in treating patients crossing the Syrian-Israeli border, the choice of treatment is influenced by the expected limited rehabilitation, lack of future follow-ups, and the unavailability of medical services in patients' home country. When soft tissue conditions are reasonable, early nerve surgery was conducted. Tendon transfers were done when the chances of nerve recovery were low
Kim et al. (2004)	118	Not provided	Upper or middle trunk	4.25 months	Posterior approach, neurolysis, sural and/or antebrachial cutaneous nerve graft	Vascular complications, wound hematoma, infection	42 months	To best maximize recovery of the individual elements, if loss in the distribution of one or more elements persists in the early postinjury months, then the patient must have an operation at which time intraoperative NAPs on lesions in continuity are performed
Kim et al. (2006)	118	Not provided	Broad spectrum of brachial plexus injuries	2 to 4 months	Neurolysis, end-to-end repair, sural and/or antebrachial cutaneous nerve graft	Pseudoaneurysm	30 months	Adult patients with lesions of C8, T1, lower trunk, or medial cord to ulnar nerve should be treated conservatively, as repair has low yield regarding ultimate functional recovery
Kline (1989)	141	28 years	Broad spectrum including root to trunk, division to cord, cord to nerve	4.25 months	Anterior approach, posterior approach, neurolysis, end-to-end repair, sural and/or antebrachial cutaneous nerve graft	Thrombosis, infection, partial loss of function	46 months	Patients selected for surgical management were those that had serious deficits. The risks of complications must be weighed seriously prior to beginning surgical intervention
Kline and Judice (1983)	46	4 years to 70 years	Roots to trunks, division to cords, cords to nerves	4.75 months	Anterior approach, neurolysis, sural and/or antebrachial cutaneous nerve graft	None provided	30 months	Delay of several months before surgical intervention is useful in guiding cases with continuity lesions to assess for potential improvements of deficits. Lesions not in continuity may be candidates for acute exploration and repair
Samadian et al. (2009)	20	20 years	Broad spectrum including root to trunk, division to cord, cord to nerve	2 months to 24 months	Neurolysis, end-to-end repair, sural nerve graft	None provided	36 months	Neurolysis of lesions in lower roots, lower trunk, and medial cord to ulnar nerve did not result in useful recovery. Surgical procedures should be selected based on the level of injury as well as the microscopic findings during the operation
Secer et al. (2009)	159	19 years to 30 years	Spinal nerve to trunk, trunk, division to cords, cords to nerve	1.5 months to 4 months	Soft tissue and vascular repair, neurolysis, sural nerve graft	Neuroma, vascular injury, osteomyelitis	6 months to 39 months	Intraoperative appearance of the nerve and type of surgery are prognostic factors of the final functional outcome of patients

## Data Availability

Data sharing is not applicable to this article as no datasets were generated or analysed during the current study.
